# Rhabdomyosarcoma and Extraosseous Ewing Sarcoma

**DOI:** 10.3390/children5120165

**Published:** 2018-12-10

**Authors:** Juan P. Gurria, Roshni Dasgupta

**Affiliations:** Cincinnati Children’s Hospital Medical Center, Division of Pediatric General and Thoracic Surgery, Cincinnati, OH 45229, USA; jpgurria@gmail.com

**Keywords:** rhabdomyosarcoma, extraosseous Ewing sarcoma, pediatric

## Abstract

Rhabdomyosarcoma (RMS) is a malignant tumor that represents the most common form of pediatric soft tissue sarcoma. It arises from mesenchymal origin and forms part of the group of small round blue cell tumors of childhood. It has a constant annual incidence of 4.5 cases per 1,000,000 children. The known histological diagnosis of the two major subtypes (embryonal and alveolar) has been recently enhanced by tumor biological markers and molecular differentiation diagnostic tools that have improved not only the updated classification based on risk stratification, but also the treatment approach based on the clinical group. Ewing sarcoma (ES) is a round cell tumor, highly malignant and poorly differentiated that is currently the second most common malignant bone tumor in children. In rare instances, it develops from an extraskeletal origin, classified as extraosseous Ewing sarcoma (EES). We provide an updated, evidence-based and comprehensive review of the molecular diagnosis, clinical and diagnostic approach and a multidisciplinary medical and surgical management according to the latest standard of care for the treatment of pediatric RMS and EES.

## 1. Rhabdomyosarcoma

Rhabdomyosarcoma (RMS) is the most common form of pediatric soft tissue sarcoma accounting for 5% of all childhood cancers [1]. It is the third most common pediatric extracranial solid tumor after neuroblastoma and Wilms tumor. RMS is a malignant tumor of mesenchymal origin and is part of the group of small round blue cell tumors of childhood which include neuroblastoma, primitive mesenchymal tumors and lymphoma. RMS incidence is approximately 350 cases each year in the United States which corresponds to approximately 4.5 cases per 1,000,000 children per year [1,2]. There is a Caucasian male predominance with a bimodal age of presentation with peaks between ages two to six years, and again between 10 and 18 years [3]. However, more than 80% of cases are diagnosed before age 14 [4]. This bimodal distribution correlates with the occurrence of the two primary histologic subtypes of RMS: Embryonal rhabdomyosarcoma (ERMS) in early childhood, which typically presents in the head, neck and genitourinary (GU) regions; and alveolar (ARMS) for the later childhood and adolescent years which is commonly located in the trunk and extremities. The incidence of the two subtypes is approximately 65–75% (ERMS) and 25–32% (ARMS). Greater than one third (35%) of RMS occurs in the head and neck followed by GU and the extremities [1,5].

The majority of cases of RMS are sporadic; however, RMS has been associated with familial syndromes, including: Beckwith-Wiedemann, Li Fraumeni, Neurofibromatosis type I (NF1) [3,6], Noonan syndrome, Costello syndrome and hereditary retinoblastoma [7].

Risk factors remain largely unstudied; however, the Children’s Oncology Group (COG) recently published that immunizations may decrease the population risk of developing RMS [8]. 

## 2. Tumor Biology, Histology and Molecular Diagnosis

RMS is felt to arise from pluripotent mesenchymal cells with disrupted and aberrant cell growth and blocked differentiation from myoblasts toward myofibers. Causal relationships have been suggested for the *MET* proto-oncogene, macrophage migration inhibitory factor (MIF), and *TP53* with regards to oncogenic transformation and tumor progression [9,10,11]. The immunohistochemical (IHC) stains used to identify RMS include MyoD1, desmin, myogenin, and muscle specific actin. ERMS is characterized by a loss of heterozygosity at the 11p15 locus in about 80% of patients. The Insulin Growth Factor II (*IGF-II*) gene lies within this locus [12], and can be overexpressed due to a paternal allele duplication. Mutations of *MYCN* and *CDK4* are more common in ARMS [13] and its appearance can mimic pulmonary parenchyma in IHC stains.

Patients with ARMS are noted to express fusion proteins arising from the fusion of the *FOXO* transcription factor gene with either *PAX3* (55%) or *PAX7* (23%) transcription factors [14,15]. In these fusions, the DNA binding domain of *PAX* is combined with the regulatory domain of *FOXO* resulting in increased *PAX* activity leading to dedifferentiation and proliferation of myogenic cells [16]. The *PAX7-FOXO* fusion results in worse overall survival (OS) [17]. 20% of ARMS cases that do not express fusion status are known as fusion-negative and have similar biologic behavior to ERMS tumors with the same loss of heterozygosity at 11p15.5 and also have comparable OS and event free survival (EFS) [18]. Studies have shown that these translocations, rather than what is seen on histopathology, appear to determine the poorer outcome noted for patients with alveolar subtype [19,20]. Fusion-positive ARMS patients are known to have a higher rate of metastatic disease compared to fusion-negative patients [21]. Alveolar histology is also associated with Myogenin expression and carries a worse prognosis [22]. Fusion status has replaced tumor histology for the classification of RMS in future treatment protocols according to the COG [21].

**Figure 1:**
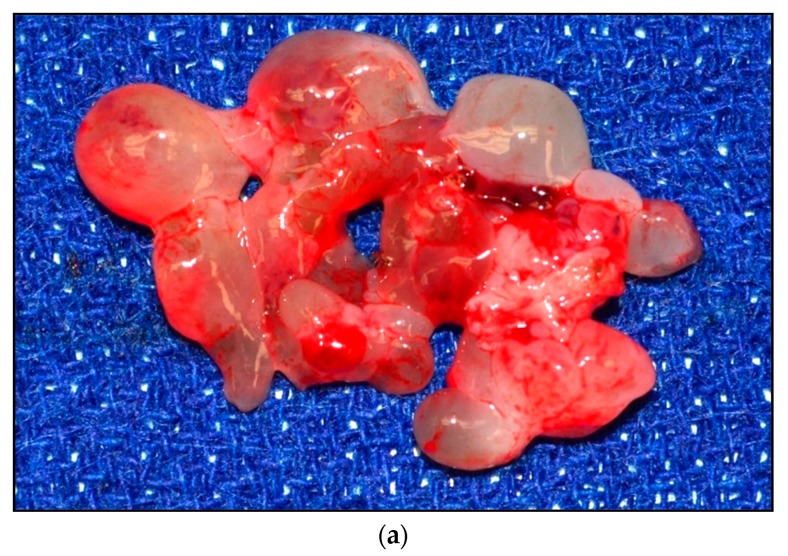

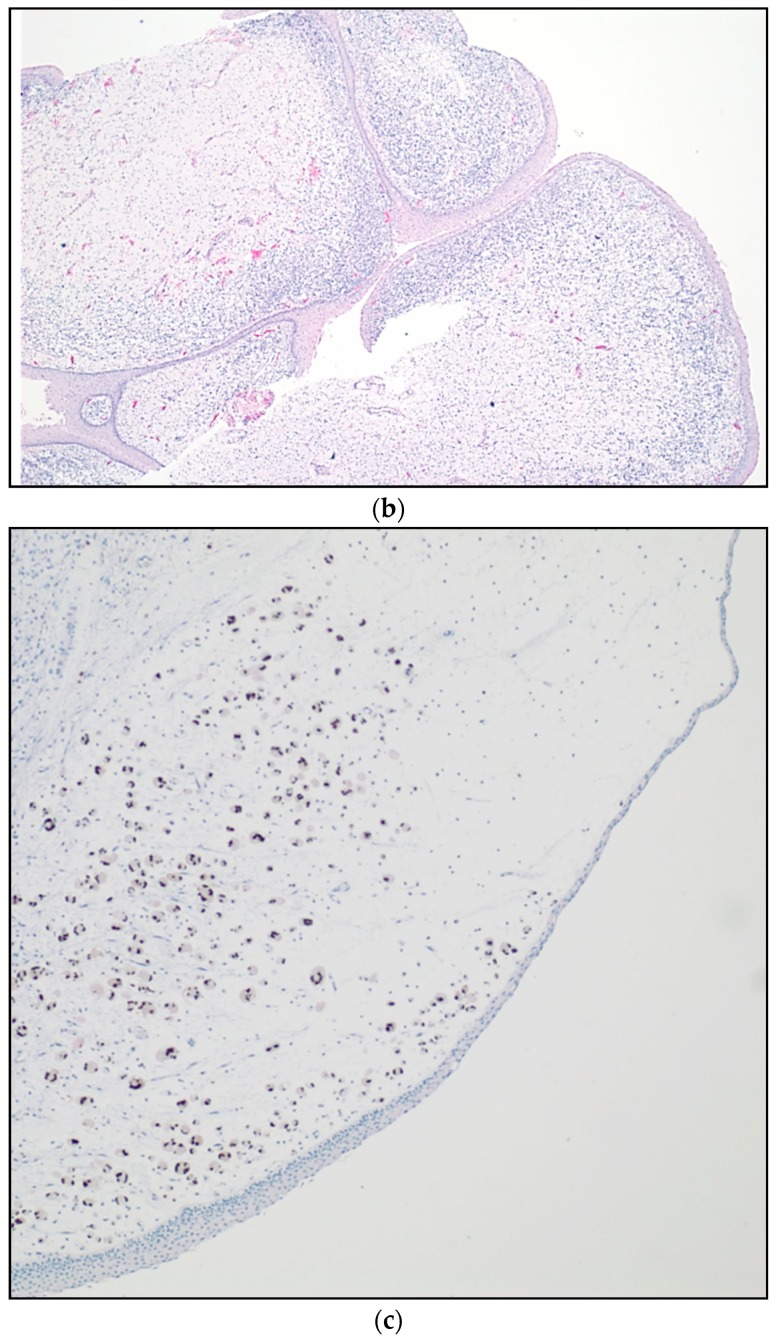
(**a**) Embryonal rhabdomyosarcoma (ERMS)—Botryoid (grape-like) gross appearance. Mucosal (and submucosal) tumor lifts up and folds the vaginal mucosa, from an infant—a classic clinical and gross presentation. (**b**) Low power histology eminently corresponds with gross botryoid appearance: Squamous non-keratinizing epithelium of vaginal mucosa covers non-demarcated ribbon-like layers of the tumor, which is relatively primitive and cellular superficially (more densely placed nuclei for a blue layer on H&E stain). (**c**) Persistent tumor post-treatment, with phenotypically “maturing” rhabdomyoblasts (larger cells with a greater amount of cytoplasm) highlighted on the immunohistochemical stain for desmin.

Historically, RMS is further subdivided according to histologic findings. ERMS includes: Botryoid, spindle cell, and dense patterns. Having the botryoid and spindle cell histology portend a better prognosis. Spindle cell histology is commonly found in paratesticular lesions while botryoid tumors are often found in hollow viscus, such as the bladder, vagina, and biliary tree [23]. ERMS is characterized by regions of loose myxoid mesenchymal tissue alternating with dense cellular regions with rhabdomyoblasts in various stages of differentiation (Figure 1a–c). The dense subtype presents with sheets of primitive cells with scant cytoplasm and ovoid nucleus, has uniformly dense cellularity superficially with prominent nucleoli and often times requires confirmatory classification by pathologists, since its easily confused with alveolar RMS [24,25]. 

**Figure 2:**
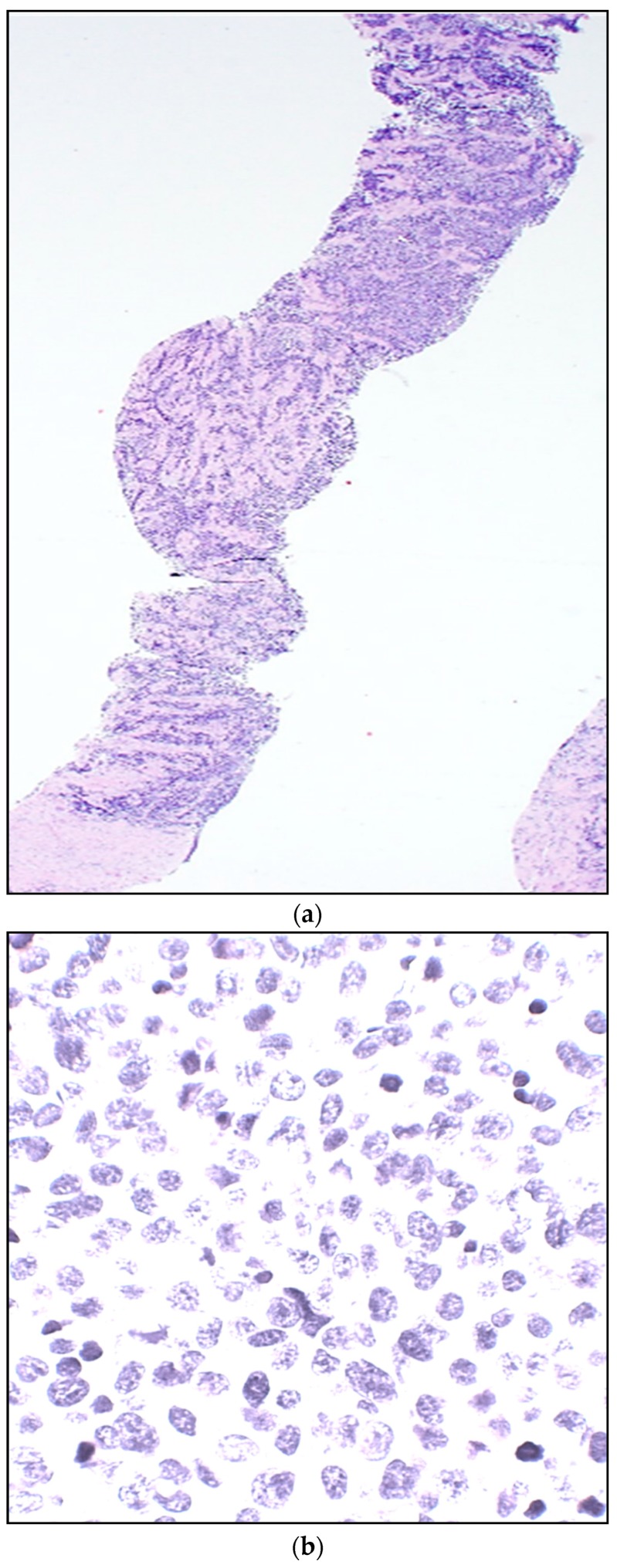

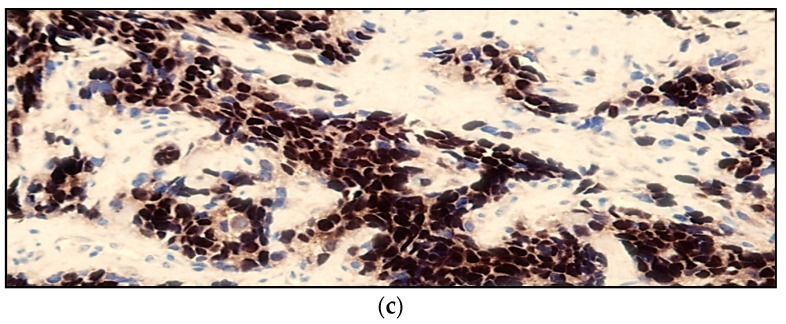
(**a**) Alveolar (ARMS)—Needle core biopsy from a soft tissue arm mass from a female teenager. A highly cellular “small round blue cell neoplasm” is seen infiltrating connective tissue in skeletal muscle. (H&E stain). (**b**) At high power, tumor cells are tightly packed, with variable molding cell membranes, small to moderate amount of delicate cytoplasm, relatively uniform round nuclei, delicate to “salt-and-pepper” chromatin, mostly inconspicuous nucleoli, and scattered mitoses (H&E stain). (**c**) Immunohistochemical stain for myogenin strongly highlights nuclei of the overwhelming majority of infiltrating tumor cells, densely packed. In contrast, nuclei of the collagenous connective tissue in the background remain appropriately negative.

ARMS is subdivided into classic and solid patterns, and by pathologic definition it requires greater than 50% of the specimen to be alveolar in nature to be classified as ARMS. Classic ARMS cells contain eosinophilic cytoplasm in nests separated with fibrous septae with islands of tumor cells, whereas the solid pattern lacks the dividing septae and is characterized by sheets of monomorphic cells with round nuclei (Figure 2a–c).

## 3. Presentation

Symptoms of RMS will depend on the location and size of the primary disease; however, patients generally present with an asymptomatic mass. Patients may also have signs and symptoms secondary to mass effect on adjacent structures and complications due to compression [26]. RMS involving the head and neck are most commonly ERMS and can present with proptosis, ophthalmoplegia, cranial nerve palsies, and meningeal symptoms [27].

Paratesticular RMS can present with painless swelling in the scrotum and is known to have a high rate of retroperitoneal lymph node metastases particularly among boys >10 years of age [28]. A recent study showed that localized paratesticular RMS has a favorable prognosis, unlike the unfavorable features, including age of diagnosis >10 years old (for which assessment of regional nodes is recommended) and tumor size over 5 cm [29]. Regional lymph node evaluation is also recommended in patients with N1 disease since it decreases EFS [29]. Tumors involving the GU tract can present with obstruction, constipation, or urinary frequency. Patients with vaginal RMS are usually younger and present with bleeding, discharge or fullness secondary to mass effect. Perineal/peri-anal RMS most commonly has alveolar histology, frequently involves regional lymph nodes, and has a poor prognosis with a five year overall survival around 45% [30,31]. Extremity RMS usually presents as a painless mass. These tumors are more aggressive and are usually ARMS [32]. Neonatal presentation of RMS is extremely rare with most cases presenting with the embryonal botryoid subtype [33].

## 4. Assessment

**Figure 3:**
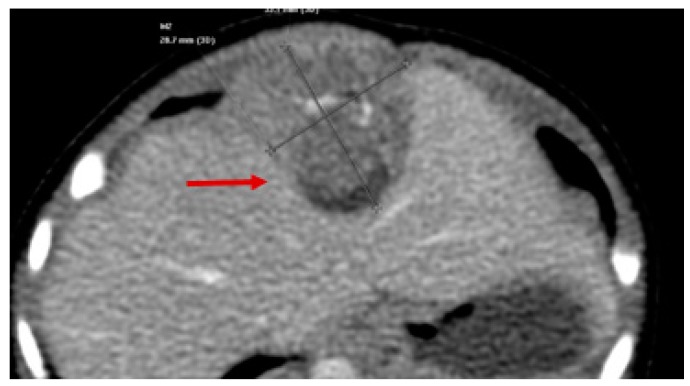
Axial CT showing Diaphragmatic Rhabdomyosarcoma (red arrow).

**Figure 4:**
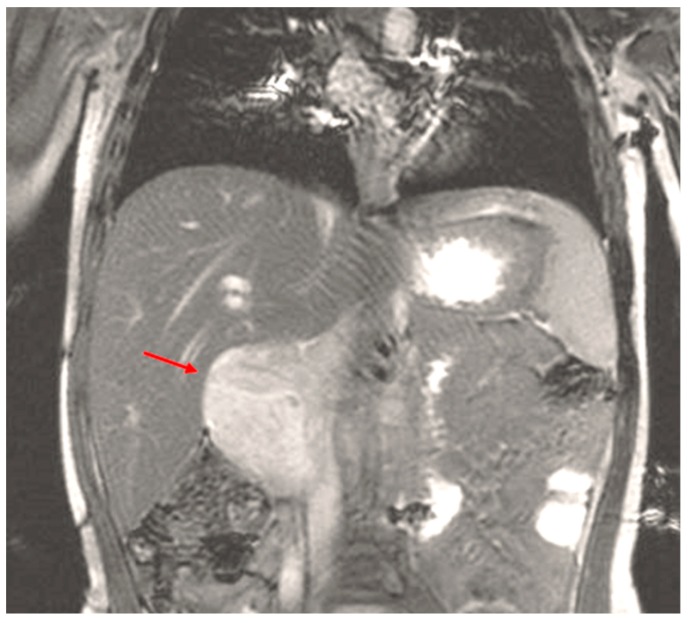
MRI (Coronal View) of patient with hepatobiliary embryonal rhabdomyosarcoma (RMS) (red arrow).

All patients with suspected RMS require a complete work-up prior to initiation of treatment, including full laboratory examination. Cross sectional imaging studies should be performed with computed tomography (CT) or magnetic resonance imaging (MRI) to assess its true size and involvement of surrounding structures or vital organs (Figure 3 and Figure 4).

For most patients, staging generally includes: Bone marrow biopsy, whole body bone scan, CT of the brain, chest for lung evaluation, and abdomen with triple phase contrast for liver assessment, and lumbar puncture for cerebrospinal fluid evaluation. However, recent studies have shown that RMS without evidence of local invasion has a low rate of metastatic disease and bone marrow biopsy and bone scan are unnecessary in these patients [34]. The use of metabolic imaging with ^18^F-Fluorodeoxyglucose positron emission tomography (FDG-PET) in the pediatric RMS population has limited experience and is not yet part of the first line imaging. It is important to note that the PET response has not correlated with patient outcomes [35,36].

## 5. Lymph Node Evaluation

Clinical and radiographic assessment of regional and distant lymph nodes should be performed in all patients prior to the initiation of treatment as this will guide staging and therapeutic management. Positive regional nodes are irradiated, and positive distant nodes are considered metastatic disease, which upstages the disease and alters therapy. Nodal disease is present in up to 25% of all RMS patients with a higher incidence in specific primary sites, such as the perineum, retroperitoneum, extremity, bladder, parameningeal, and paratesticular. Positive lymph nodes are also an independent poor prognostic factor for OS and failure free survival (FFS) in patients with fusion positive ARMS. This does not appear to be an adverse prognostic factor for fusion negative ERMS patients, provided they undergo radiation therapy (RT) [37]. Current indications for nodal evaluation include positive clinical nodes, defined as >1 cm by CT or MRI, or 18-FDG avid (N1), extremity, trunk and paratesticular tumors in patients >10 years old, and is recommended in all patients with fusion positive ARMS given its poor outcome and high incidence of false negative imaging [38,39]. In as yet unpublished data for patients >10 years old with paratesticular RMS (known to have improved survival when lymph node sampling is performed) [39], there will be a recommendation from the COG to transition to sampling nodes rather than performing a template dissection, due to the long term adverse effects and taking at least six nodes from the iliacs and para-aortic region. 

**Figure 5:**
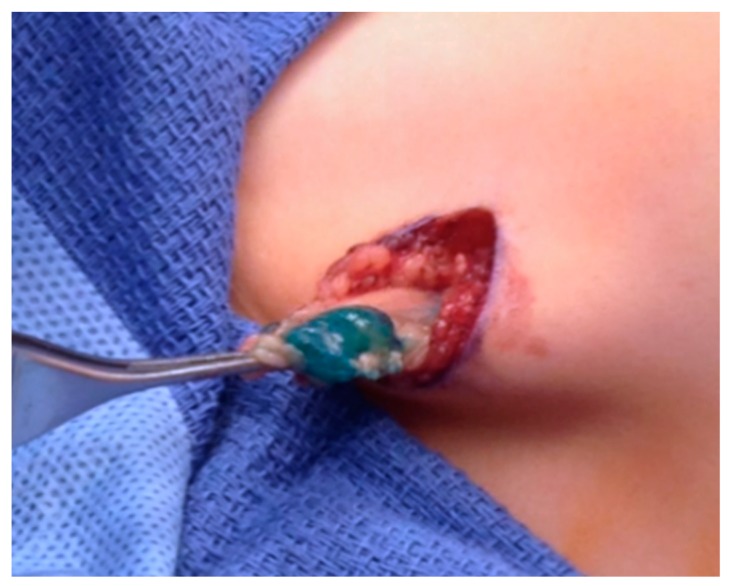
Axillary Sentinel Lymph Node Biopsy for trunk RMS. Blue dye can be appreciated in the sample.

Biopsy is necessary to confirm local and disseminated metastatic disease. In the absence of palpable nodes, sentinel lymph node (SLN) biopsy is the technique of choice and should be performed to assess involvement, particularly for patients with extremity or trunk RMS (Figure 5). Technetium 99 labeled tracers and Isosulfan blue dye are the most common agents used to complete SLN biopsy.

Completion lymph node dissection is unnecessary and does not improve outcome [32]. Extremity tumors with in transit nodes require aggressive evaluation since the incidence of involvement is higher than anticipated, and failure to include these nodal group in the radiation field increases the chances for local and regional tumor failure [40]. Laparoscopy for retroperitoneal lymph node dissection could be achieved; however, should be done by experienced surgeons only.

## 6. Staging

Staging of RMS follows the classic TNM classification and defines a pretreatment system determined by site and size of the primary tumor, degree of invasion, nodal status, and the presence or absence of metastatic disease. It is based on the preoperative physical examination and imaging studies (Table 1).

## 7. Clinical Group

One of the most important prognostic factors in RMS is the extent of residual disease after initial resection; this underscores the importance of a complete surgical resection if possible. After the initial surgical procedure, the patients are assigned to a clinical group according to the pathologic evaluation of the specimen, which encompasses the completeness of excision and evidence of tumor metastasis to lymph nodes or distant sites. The clinical group assigned refers to the pathologically determined extent of the tumor after resection or biopsy of the primary lesion, along with the lymph node evaluation and the patient’s status prior to the initiation of systemic therapy (Table 2).

## 8. Risk Group Stratification

The Soft Tissue Sarcoma Committee of the Children’s Oncology Group (STS-COG) created the risk stratification system in an effort to tailor therapy to patient outcomes. It incorporates pre-treatment staging (including site and TNM status), the extent of disease after surgical resection (clinical group), and histology/fusion status into a more comprehensive classification of patients into: Low, intermediate, and high-risk groups. This comprehensive stratification process has shown to be an accurate predictor of outcomes (Table 3).

The European Study groups International Society of Paediatric Oncology-Malignant Mesenchymal Tumor and Cooperativee Weichteilsarkom Studiengruppe (SIOP-MMT and CWS) established a similar risk-based classification based on prognostic factors (Table 4).

## 9. Treatment

### 9.1. Medical Treatment

Systemic therapy is the primary backbone of a treatment plan for all RMS patients and its composition is based on risk stratification. The standard chemotherapy regimen includes: Vincristine, actinomycin-D and cyclophosphamide (VAC). For the low risk group (LRRMS), the duration of chemotherapy and the dosing of cyclophosphamide can both be decreased from the current regimen dosing while maintaining good outcomes, thereby limiting its toxicity [41]. Irinotecan (I) was added in a recent randomized control trial to the intermediate risk (IRRMS) group in the form of VAC/VI since it has shown a significant benefit with metastatic and recurrent RMS. Although no improvement in EFS, or overall survival (OS) compared to VAC alone was noted, there was a lower rate of toxicity and cumulative dose of cyclophosphamide in the VAC/VI regimen. This toxicity data supports its use as the current standard therapy for RMS [42]. A recent study, however, had different results when decreasing total Cyclophosphamide dose for patients with subset 2 low-risk RMS that did not receive RT. These patients had a decrease in FFS and an increase in local recurrence when the total dose of Cyclophosphamide was decreased [43]. For patients with high risk disease, slow progress is being made in the development of prospective trials despite the use of new chemotherapeutic agents and molecular therapies [44,45]. A recent multicenter, open-label randomised, controlled, phase 3 trial was performed in 14 European countries and over 100 hospitals comparing the current standard chemotherapy (IVA) to the same standard (IVA) plus a dose-intensified doxorubicin regimen for patients with high-risk non-metastatic RMS; and despite being doxorubicin an effective drug against RMS, added to a multidrug regimen in this case, failed to significantly improve the outcomes in these specific cohorts of patients in Europe [46]. The current intermediate risk trial ARST 1431 is comparing standard chemotherapy vs. standard chemotherapy and temsirolimus in treating patients with IRRMS hypothesizing that temsirolimus (m-tor inhibitor) may improve survival in conjunction with standard chemotherapy [47]. Approximately 20–30% of patients with localized RMS that have undergone standard therapy and achieve remission will present with relapse, which confers a poor survival. A recent European study evaluated whether the addition of maintenance low-dose chemotherapy in patients with high-risk RMS in complete remission after standard therapy would improve survival, and showed a significant increase in EFS (78% vs. 72%) and OS (87% vs. 77%) by adding 6, 28-day cycles of vinorelbine on day 1,8,15 of each cycle and continuous daily oral cyclophosphamide [48].

Significant advances have been made in regard to reducing therapeutic dosing and timing for chemotherapeutic agents and radiotherapy when appropriate; however, in specific cases this approach is not recommended. A recent prospective trial showed excessive locoregional treatment failures in patients with parameningeal RMS when reduced-dose cyclophosphamide and delayed radiotherapy was attempted [49].

### 9.2. Radiotherapy (RT)

Except for Clinical Group I ERMS, RT along with surgical resection are essential parts of a local control portion of the treatment plan. The anatomic location, extent of residual disease after surgical resection, and lymph node involvement will dictate dosing and timing of therapy [50]. RT is given between 6 to 12 weeks after beginning of chemotherapy except for patients with para-meningeal RMS with intracranial extension, in which an earlier start provides better local control outcomes [27]. RT dosing ranges between groups: Group I ARMS (36 Gy), Group II (41.4 Gy) and Group III (50.4 Gy) [51]. Studies have shown that conservative surgery plus brachytherapy can also conserve vital structures and function without compromising outcomes [22]. Unlike children and adolescents, infants are a significant challenge secondary to long-term toxicity which can include facial growth retardation, neuroendocrine dysfunction, visual/orbital problems, hearing loss, hypothyroidism, developmental delay, esophageal stenosis, leukemia, and brain hemorrhage [52,53]. Current strategies are targeting local control with intensity modulated radiation therapy (IMRT) and proton beam RT, which can avoid under-treatment while providing more focal treatment with decreased adverse effects [27]. However, de-escalation of effective therapies due to concerns from treatment toxicity should be considered cautiously, given an increase in local recurrence seen in a current review of infants with localized RMS treated with individualized local therapy compared to patients who received protocol-specific local therapy (local failure rate 35% vs. 16%, *p* = 0.02) [54]. A recent large prospective cohort showed that proton RT represents a safe and effective radiation modality for pediatric RMS patients with improved five-year local control (81%), EFS (69%) and OS (78%) [55]. 

### 9.3. Primary Surgical Resection

All patients with suspected RMS need a thorough surgical evaluation since local surgical control is an important determinant of outcome. Local recurrence is the most common reason for treatment failure for patients with localized disease. For tissue diagnosis, open biopsy is generally recommended to obtain enough tissue for biology and chromosomal analyses. If core needle biopsies are performed, multiple passes are needed to avoid sampling error and ensuring enough tissue is available for biologic studies.

Complete surgical excision if possible should be performed initially as long as there will be no major functional impairment or disfiguring cosmetic result. This is particularly challenging in sites, such as the orbit, bladder, prostate, vagina, and uterus. The goal is to achieve complete resection with normal tissue margins of at least 0.5 cm surrounding the tumor. Margins should be marked and oriented for adequate histopathologic review. Debulking procedures are generally not indicated [56]. In cases where complete excision is not possible and residual disease is left, the surgical bed should be marked with small titanium clips to guide RT and future re-excisions if needed. Tumors that are removed piecemeal are considered Group II even if all gross tumor is removed.

### 9.4. Pre-Treatment Re-Excision

Pre-treatment re-excision (PRE) of RMS should be considered in cases where the surgical margins are positive, a non-oncologic excision was performed, or when only a biopsy was taken, if the surgeon feels that a complete resection with negative margins is feasible prior to starting chemotherapy. It is generally performed for extremity and trunk RMS. 

Patients who undergo PRE and achieve negative margins are then categorized as Group I, and have the same outcome as patients with negative margins following initial excision. The use of PRE has shown to improve FFS and OS [57].

### 9.5. Delayed Primary Excision (DPE)

Response to therapy is generally evaluated with CT/MRI after induction chemotherapy around week 12, but it has been shown that it does not correlate with outcome [58]. However, pathologic response has a direct association with prognosis. In a reported series, 79% of pathology specimens after DPE contained viable tumor after 12 weeks of systemic therapy and had lower FFS rates [59]. A delayed primary excision (DPE) should be considered in patients with residual disease after chemotherapy, if a complete resection can be achieved without significant morbidity. The goal of this DPE is to achieve local control, thereby reducing the RT dose and the associated morbidity. Recent studies propose tailoring RT dosing based upon completeness of excision (36 Gy for complete excision, 41.4 Gy for microscopic residual disease, and 50.5 Gy for gross residual disease). One study showed that almost 75% of patients with IRRMS were eligible for a dose reduction without compromising local control compared to historical controls [59,60]. DPE should not be mistaken for resection of residual masses after completion of standard therapy. Second look operations (SLO) should be considered for local control, during or after adjuvant therapy for tumors that are initially unresectable, but show a significant response to induction chemotherapy and RT and can be completely resected [51].

## 10. Metastatic Disease and Recurrent RMS

**Figure 6:**
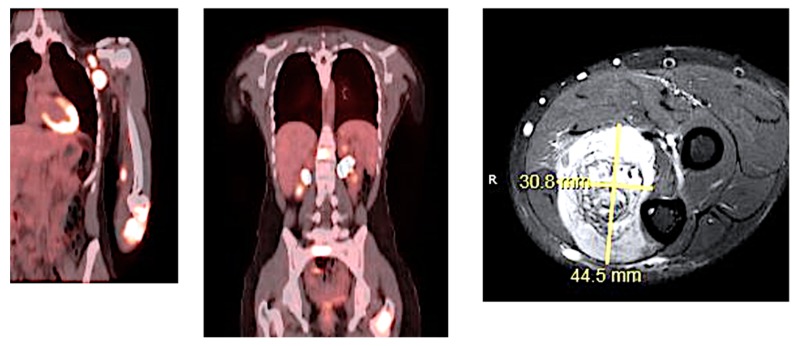
PET/CT and MRI of 19 y/o patient with Stage IV extremity alveolar RMS. Uptake can be appreciated in the left axillary node, left elbow and left femoral head.

**Figure 7:**
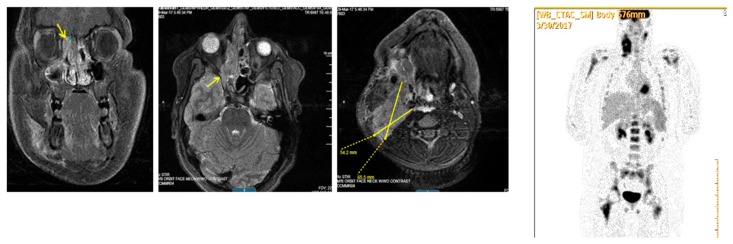
MRI T-1 weighted image of 13 y/o patient with Stage IV parameningeal alveolar RMS. (**Left**) image shows in a coronal plane a heterogeneous mass centered within the right ethmoid air cells (arrow) and superior nasal cavity mildly displacing the orbit. (**Right**) image—axial cut shows right neck lymphadenopathy shown as well with high uptake in PET scan image at the far right.

**Figure 8:**
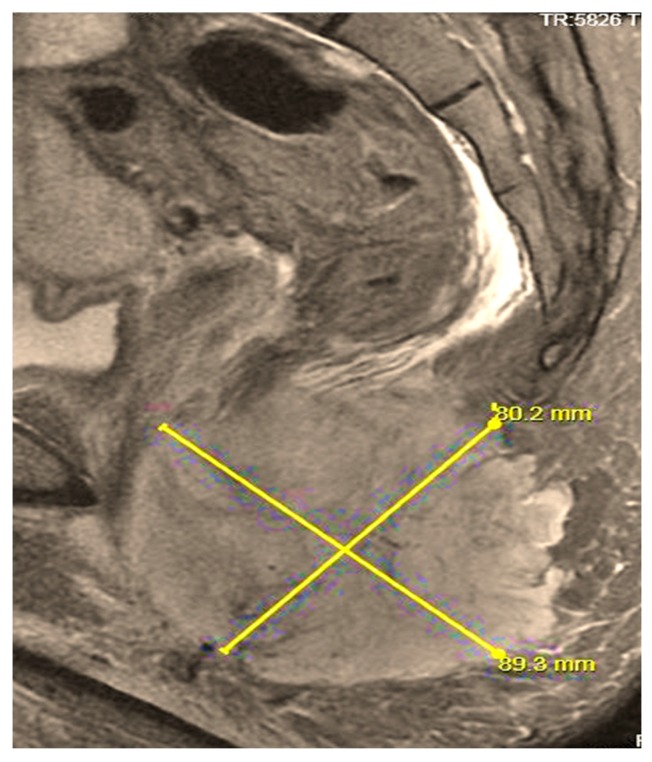
MRI (sagittal view) of 15 y/o patient with Stage IV perineal alveolar RMS.

RMS can metastasize via both hematogenous and lymphatic routes. Metastatic disease is associated with a very poor prognosis and has a 5-year survival under 20%. Patients who initially present with metastatic disease and/or have gross residual disease in unfavorable sites after their initial surgery, are also more likely to have recurrent disease. Early relapse is associated with a poor prognosis; there is evidence that resection of recurrent RMS can improve five year survival from 8% to 37% [61]. Pre-clinical trials showed promising results for agents, including: Trabectedin, Cixutumumab and Temsirolimus; however, in recent clinical trials, these agents have failed to demonstrate sufficient clinical activity against relapsed RMS [62]. New treatments include the addition of Bevacizumab and Temsirolimus to VAC for salvage chemotherapy. (Figure 6, Figure 7 and Figure 8).

## 11. Outcomes

Overall, the patient’s age, site and size of primary tumor, clinical group, histopathology with fusion status, +/- regional lymph node involvement, and the presence, or absence, of metastasis drive the prognosis for RMS. As described previously, favorable characteristics include age <10 years at diagnosis, tumor size less 5 cm, embryonal fusion negative tumor, orbit and non-parameningeal head/neck primary, tumor completely excised prior to initiation of chemotherapy, and the lack of metastatic disease at diagnosis [22]. Group I low risk patients have >90% survival even with reduced chemotherapeutics dosing. Group II patients with microscopic residual disease have an 85% survival. In this group, however, patients with alveolar histology, unfavorable primary sites, and nodal involvement have the worst outcomes [63]. Group III patients have increased FFS in patients with tumors <5 cm, in favorable sites, and without lymph node involvement [64]. A recent report by the European Paediatric Soft Tissue Sarcoma Study Group (EpSSG) studied the outcomes of fusion status in 103 patients with alveolar RMS presenting with N1 disease, and showed that patients with positive fusion genes for forkhead box protein O1 (FOXO1) appear to have worse prognosis and affected the five-year EFS for these patients (43%) compared with alveolar RMS N1 fusion-negative patients (74%) in this cohort [65].

New strategies are being used to potentially increase OS for patients with metastatic RMS and peritoneal sarcomatosis. Hyperthermic intraperitoneal chemotherapy (HIPEC) is currently offered in select centers in addition to chemotherapy and RT as a salvage therapy for advanced RMS and sarcomatosis with complete cytoreduction; however, results are still preliminary [66].

Patients with a history of RMS have increased risk of secondary malignancies and have increased mortality compared to the general population [67]. All children treated for RMS should be monitored regularly with physical examinations and imaging every 3–6 months for the first two years after completing treatment. All physicians should follow growth patterns, sexual maturity, fertility, and signs and symptoms of recurrence, including: Weakness, weight loss, neurological deficits, bone pain, bleeding, and recurring infections.

## 12. Extraosseous Ewing Sarcoma

Ewing sarcoma (ES) is a round cell tumor, highly malignant and poorly differentiated that is currently the second most common malignant bone tumor in children [68]. In rare instances, it develops from an extraskeletal origin, classified as extraosseous Ewing sarcoma (EES). It is usually found in older children and adolescents compared to Ewing sarcoma from bone origin in the pediatric population. EES is also present in a bimodal distribution and is more commonly found in patients less than five years old and older than 35 compared with ES [69]. It is morphologically indistinguishable from classic Ewing sarcoma and must be distinguished from the group of small round blue cell tumors [70]. Extraskeletal involvement includes: Head and neck, retroperitoneum, omentum, paravertebral, orbits, skin, chest wall, pelvis and lower extremities [71]. EES has also been reported to arise in the gastrointestinal tract, including: Pancreas, omentum, kidney and adrenals [72]. Less than 5% of EES arise in the maxillofacial region, it is usually seen in the first two decades of life and has reported better prognosis along with head and neck compared to other sites [73]. Several studies from small series to multicenter trials have proven that outcomes for patients with EES are at least similar to that of Ewing sarcoma of the bone [74]. 

## 13. Tumor Biology and Histology

Ewing sarcoma shares with peripheral primitive neuroectodermal tumor (pPNET) a balanced translocation t(11;22) (q24;q12) in over 85% of cases; however, they comprise a different spectrum of the same histological group of neoplasms encompassed in the term Ewing sarcoma family tumor (ESFT) [75,76]. ESFT is composed of tumors associated with specific chromosomal translocations that lead to the fusion of the 5’ segment of the *EWS* gene on chromosome 22, with the 3’ segment of genes of different chromosomes of which the most common is 11. This t(11;22) (q24;q12) translocation creates a fusion with the *FLI-1* gene [77], which can be detected with polymerase chain reaction (PCR) or fluorescence in situ hybridization (FISH). The origin of these tumors has been thought to be from neuroectodermal and mesenchymal cells; however, it is currently believed that they originate from human marrow mesenchymal stem cells (MSC) [78]. MSC can initiate reprogramming towards ES cancer cells through the characteristic *EWS-FLI-1* fusion [79]. 

The *EWS-FLI-1* fusion protein promotes an impaired transcriptional regulator whose suppression in ESFT cell lines results in decreased tumor formation [80]. There have been current molecular studies that show the alterations in the regulation of damage-induced transcription from the resultant fusion proteins in the Ewing sarcoma cells. These alterations accumulate R-loops and promote increased replication stress. They also create increased interaction between *BRCA1* and the elongating transcription machinery that impair the homologous recombination [81]. 

Histology of tumors in this family is currently classified as “classical”, or “variant” [82]. Classical ES is the prototypic undifferentiated small round cell tumor characterized by round oval sheet cells with primitive nuclei and clear cytoplasm. Atypical histology is characterized by large and pleomorphic cells with irregular nuclear membranes with clear eosinophilic cytoplasm. There are numerous variant histological types with distinct morphologic features that classify them into: Large cell, pPNET, adamantinoma-like, spindle cell sarcoma-like, sclerosing and vascular-like [83].

Immunohistochemistry has improved the diagnosis and classification of ESFT through the description of markers associated with these tumors. These markers include CD99, FLI-1, and caveolin1, which helps in the differentiation from other small round cell tumors [83]. Other cell surface glycoproteins include MIC2 surface antigen encoded by the CD99 (*MIC2X*) gene which is a sensitive diagnostic marker of ESFT [84]. Electron microscopic features include a specific high nucleus to cytoplasm ratio with aggregated glycogen granules in the cytoplasm [82]. Molecular genetics should be mandatory for diagnostic accuracy of ESFT and other sarcomas to guide clinical management even when a histological diagnosis is made by expert pathologists [85].

Differential diagnosis includes other small round blue cell tumors involving bone and soft tissue, including: Lymphoma, small cell osteosarcoma, mesenchymal chondrosarcoma, undifferentiated neuroblastoma, synovial sarcoma, desmoplastic small round cell tumors and rhabdomyosarcoma. Key in the differential diagnosis is also the subset of tumors classified as undifferentiated small round cell sarcomas, which comprise a class of highly aggressive *EWSR1*-negative sarcomas also referred to as “Ewing-like tumors”. These tumors can present with unique gene fusions, including: *CIC-DUX4*, *BCOR-CCNB3* or *CIC-FOXO4* that can harbor several different translocations that will result in both bone and soft tissue tumors [86]. The characteristics and presentations of these tumors are complex and not part of the scope of this chapter.

## 14. Assessment

Age at initial diagnosis ranges from 2.5 to 17 years in some series. Clinical manifestation of EES varies largely depending on the site of origin, although the most classic presenting symptom is a rapidly growing mass producing pain.

**Figure 9:**
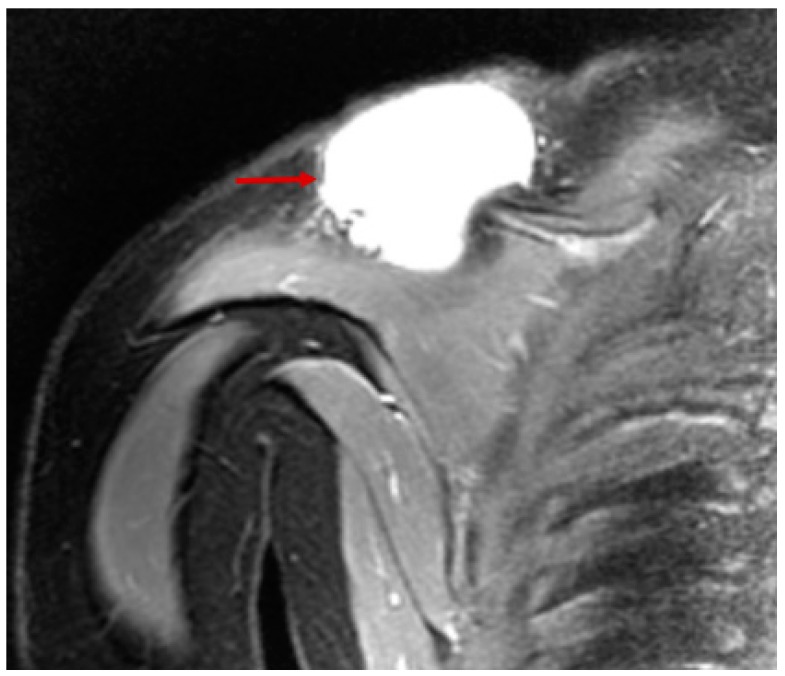
Coronal MRI of extraosseous Ewing sarcoma. Note cystic nature of lesion on T2 weighted image.

**Figure 10:**
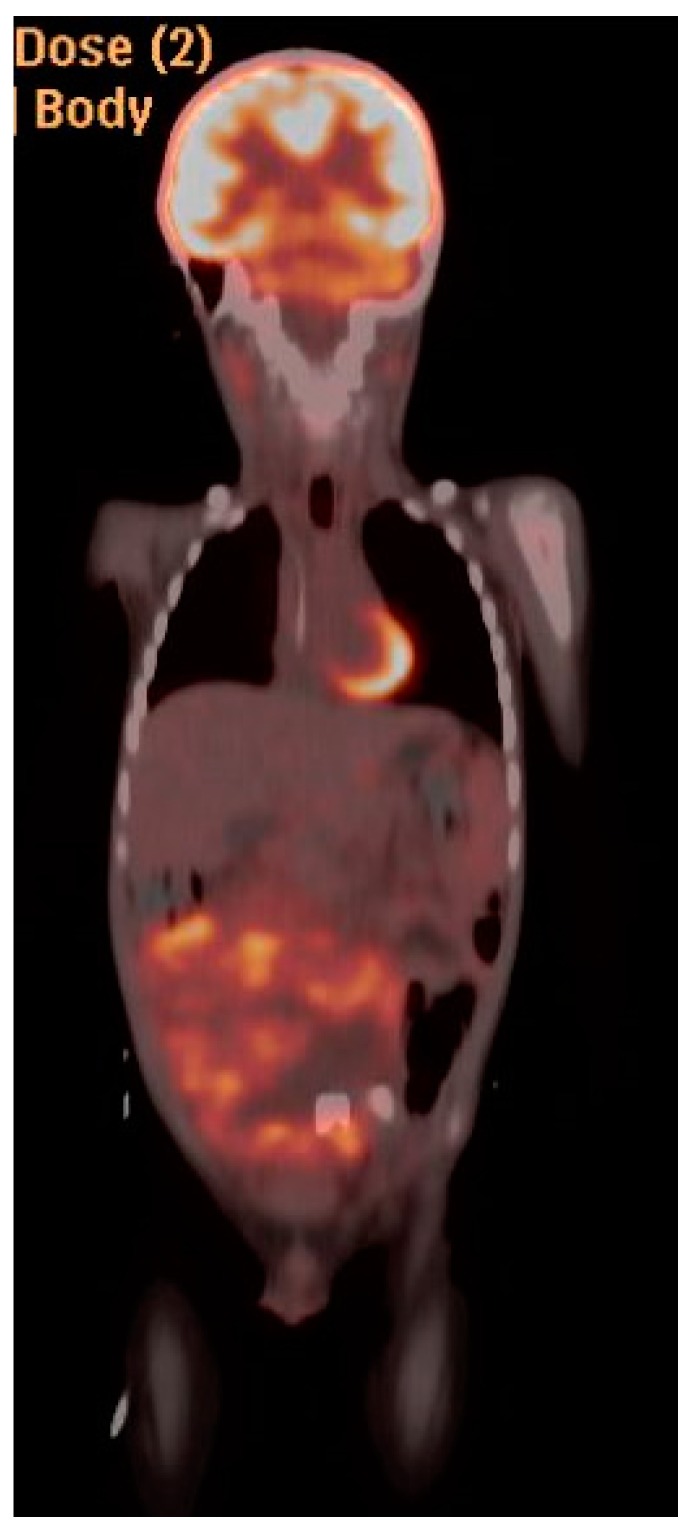
PET CT coronal view of intraabdominal Ewing sarcoma.

Initial imaging is usually ultrasonography with extra-axial presentation which usually shows a well delineated mass with low echogenicity and intratumoral vascular flow signals [87]. Cross-sectional imaging with computed tomography (CT) or magnetic resonance imaging (MRI) should follow these findings. CT usually shows a sharply delineated mass of equal density compared to muscle. MRI often shows a mass of low to intermediate signal intensity on T1-weighted images and of high signal intensity on T2-weighted images with heterogeneous contrast enhancement [87] (Figure 9 and Figure 10).

Diagnosis is made with CT-guided core-needle biopsy or pathological evaluation of the specimen after surgery. Confirmatory studies follow with immunohistochemistry and tumor markers [88]. 

Extraosseous Ewing sarcoma tends to grow along tissue planes and rarely violate major fascial planes or bone. It can form a pseudocapsule and compress surrounding tissues. The most common form of spread is hematogenous, primarily to lungs. The incidence of distant metastatic disease at the time of diagnosis is reported at 10%, of which over 80% are located in the lungs [89]. The presence of metastasis at diagnosis has been consistently the strongest adverse clinical prognostic factor [90], along with larger tumor size and older age [91].

Metastatic workup is mandated for EES as with any other malignancy and should include: Bone marrow biopsy, whole body bone scan, CT of the brain, chest for lung evaluation, and abdomen with triple phase contrast for liver assessment, lumbar puncture for cerebrospinal fluid evaluation, bone marrow biopsy, ± screening MRI of spine and pelvis. It is important to also include cytogenetics and molecular studies and lactate dehydrogenase level. Fertility consultation is advised.

Staging follows both the National Comprehensive Cancer Network (NCNN) and the Surgical Staging System from the Musculoskeletal Tumor Society. 

## 15. Medical Treatment

Treatment is usually over 6–9 months with alternating courses of chemotherapy regimens of (1) vincristine, doxorubicin and cyclophosphamide and (2) ifosfamide and etoposide. Multiagent chemotherapy for at least nine weeks prior to local therapy is advised by NCCN guidelines [92]. A recent Children’s Oncology Group (COG) report showed that chemotherapy interval compression from three to two weeks improves outcomes without increased toxicity [93]. Chemotherapy is a key element in the treatment of EES; both neoadjuvant and adjuvant therapy have shown comparable results in patients with localized disease [94]. Chemotherapy improves overall survival (OS) and reduces the incidence of recurrence [95]. Neoadjuvant chemotherapy with subsequently delayed resection has shown to increase the likelihood of complete resection with negative microscopic margins for chest wall ES/pPNET tumors and avoid radiation [96]. 

Current regimens include a combination of vincristine, cyclophosphamide, actinomycin D and doxorubicin. Further studies have shown that incorporating ifosfamide and etoposide as second-generation regimens improves disease-free survival for patients with localized disease [97]. A study showed that ESFT managed by multiagent neoadjuvant chemotherapy improved overall outcomes and local disease control close to 90% at 10 years [98]. A current retrospective review showed no significant differences between anthracycline and platinum based regimens in regards to event-free survival (EFS) and OS in adolescents [99].

Currently, the recommended regimen includes alternating vincristine, doxorubicin, cyclophosphamide and ifosfamide, etoposide cycles, along with local tumor treatment with surgical resection and/or radiation therapy [93].

Novel medical therapies to both RMS and Ewing’s are currently being developed that target specific fusion protein formation and interaction with replication. One recent study evaluated the combination effects of the histone deacetylases inhibitor suberoyalinilide hydroxamic acid (SAHA) and Lysine-specific demethylase 1 inhibitor (HCI-2509) on several biological functions in ES proving that epigenetic drugs can potentially inhibit the *EWS-FLI1* fusion protein and tumor growth in ES [100]. 

## 16. Local Control Therapy

Treatment of EES should follow a multidisciplinary approach integrated by systemic and local therapies. Surgery plays likely the most important role in EES compared to ES given that complete resection has historically shown to be a predictor of favorable survival [101]. The extents of negative margins have shown not to influence local control and the goal of surgical resection is to achieve a three-dimensional tumor-free margin [102]. Patients without negative margins at resection, however, have decreased disease-free survival [103]. 

Extraosseous Ewing sarcoma is a radiosensitive tumor; however, recent studies have recommended weighing the morbidity associated with radiotherapy compared to the one of surgical therapy when local control is attempted [104]. There is a reported dose-response effect for ESFT to radiotherapy between <40 Gy and >40 Gy that provides statistically significant improved local control when patients are grouped by tumor size. Smaller tumors showed improved local control regardless of radiation dose, which supports the use of dose-attenuated radiotherapy [104]. Radiotherapy improves local control; however, has had alternating results in regards to OS and the role of adjuvant local radiotherapy after complete resection is still inconclusive [71].

## 17. Outcomes

Overall, the prognosis of EES is more favorable compared to ES [69]. In a series of 192 cases the overall survival (OS) at five years was 75%, event-free survival (EFS) at five years was 68% for metastatic patients compared to 83% for nonmetastatic patients [74]. A study showed that the strongest predictor of favorable OS was age <14 years at diagnosis [104]. The COG recently reported that patients with extraskeletal primary tumors were more likely to have an axial primary site, less likely to have large primary tumors, and had a statistically significant better prognosis than did patients with skeletal primary tumors [105]. Relapse rates are as high as 30% in some series and most patients will not survive despite attempts of salvage chemotherapy. Tumor size over 8 cm, metastatic disease at diagnosis and relapsed disease have the worst outcomes [106].

## Figures and Tables

**Table 1:** Pretreatment Staging.

Stage	Sites	T	Size	N	M
1	Orbit, head and neck ^a^, genitourinary ^e^, Biliary tract	T1 or T2	A or B	N0 or N1 or Nx	M0
2	Bladder/prostate, extremity, cranial, parameningeal, other ^o^, non-Biliary tract	T1 or T2	A	N0 or Nx	M0
3	Bladder/prostate, extremity, cranial, parameningeal, other ^o^, non-Biliary tract	T1 or T2	A B	N1 N0 or N1 or Nx	M0 M0
4	Any	T1 or T2	A or B	N0 or N1	M1

Sites: ^a^ no parameningeal involvement; ^e^ no bladder or prostate involvement; ^o^ trunk, retroperitoneum, thoracic; Size: A, ≤5 cm in diameter; B, >5 cm in diameter; T, tumor: T1, confined to site of origin; T2, extends into surrounding tissue; N, nodes; N0, no lymph node involvement; N1 clinically involved lymph nodes defined as >1 cm by CT or MRI, or 18-FDG avid; Nx, unknown lymph node status; M, metastasis; M0, no metastasis; M1, metastasis present.

**Table 2:** Clinical Group for Rhabdomyosarcoma.

Group	Definition
Group I	Complete resection of localized disease
Group II	Regional resection
IIA	Regional resection Inadequate microscopic margins
IIB	Regional resection Lymph node disease Adequate microscopic margins
IIC	Regional resection Lymph node disease Inadequate microscopic margins
Group III	Incomplete resection Biopsy only
Group IV	Distant metastatic disease

**Table 3:** Risk Group Stratification for Rhabdomyosarcoma.

Risk	Stage	Group	Histology	Overall Survival
Low, Subset 1	1 or 2 1	I or II III or bit	ERMS ERMS	90%
Low, Subset 2	1 3	III non-orbit I or II	ERMS ERMS	
Intermediate	2 or 3 1, 2, or 3	II or III I, II or III	ERMS **ARMS**	60–80%
High	4	IV	ERMS or **ARMS**	20–40%

ERMS = Embryonal Rhabdomyosarcoma; ARMS = Alveolar Rhabdomyosarcoma.

**Table 4:** Risk Stratification for Rhabdomyosarcoma according to European Studies. Stage IV is excluded.

Metastasis	N-Status	Pathology	IRS-Group	Localization	Size/Age	Subgroup	Risk Group
M0	N0	ERMS	I	Any	≤5 cm and <10 years	A	Low
					>5 cm or ≥10 years	B	Standard
			II, III	ORB, UG-non BP, HN-non PM	Any	C	
				EXT, UG-BP, HN-PM, others	≤5 cm and <10 years	D	
					>5 cm or ≥10 years	E	High
	N1	ERMS	II, III	Any		F	
	N0	ARMS	Any			G	
	N1	ARMS	Any			H	Very High

ORB = Orbit; UG non BP = Genitourinary non-bladder or prostate tumor; HN-non PM = Non-parameningeal head and neck tumor; HN-PM = Parameningeal tumor; UG—BP = Genito-urinary bladder or prostate; Ext = Extremities.
